# Characterization of Affective Behaviors and Motor Functions in Mice With a Striatal-Specific Deletion of *Bmal1* and *Per2*

**DOI:** 10.3389/fphys.2022.922080

**Published:** 2022-06-08

**Authors:** Konrad Schoettner, Mariana Alonso, Margo Button, Cassandra Goldfarb, Juliana Herrera, Nour Quteishat, Christiane Meyer, Andreas Bergdahl, Shimon Amir

**Affiliations:** ^1^ Department of Psychology, Center for Studies in Behavioral Neurobiology, Concordia University, Montreal, QC, Canada; ^2^ Department of Health, Kinesiology and Applied Physiology, Concordia University, Montreal, QC, Canada

**Keywords:** clock genes, medium spiny neurons, mood-and anxiety-like behavior, motor coordination, mitochondrial respiration

## Abstract

The expression of circadian clock genes, either centrally or in the periphery, has been shown to play an integral role in the control of behavior. Brain region-specific downregulation of clock genes revealed behavioral phenotypes associated with neuropsychiatric disorders and neurodegenerative disease. The specific function of the clock genes as well as the underlying mechanisms that contribute to the observed phenotypes, however, are not yet fully understood. We assessed anxiety- and depressive-like behavior and motor functions in male and female mice with a conditional ablation of *Bmal1* or *Per2* from medium spiny neurons (MSNs) of the striatum as well as mice lacking one copy of *Gpr88*. Whereas the conditional knockout of *Bmal1* and *Per2* had mild effects on affective behaviors, a pronounced effect on motor functions was found in *Bmal1* knockout mice. Subsequent investigation revealed an attenuated response of *Bmal1* knockout mice to dopamine receptor type 1 agonist treatment, independently of the expression of targets of the dopamine signaling pathway or mitochondrial respiration in MSNs. The study thus suggests a potential interaction of *Bmal1* within the direct dopamine signaling pathway, which may provide the link to a shared, MSN-dependent mechanism regulating affective behavior and motor function in mice.

## Introduction

The circadian system in mammals comprises a multi-oscillator network of biological pacemakers to enable temporal coordination of biochemical, physiological, and behavioral processes relative to the 24-h environment. The role of the master circadian clock located in the suprachiasmatic nucleus (SCN) is primarily understood in conveying external time to clocks located in other brain areas and tissues, which utilize the SCN-derived time cues to control tissue-specific processes according to the respective time of day. The hierarchical system thus provides temporal integrity to the entire biological system in a daily changing environment to maintain systemic homeostasis required for its optimal function and performance (de Assis and Oster, 2021).

At the cellular level, daily oscillations are driven by the rhythmic expression of circadian clock genes. The circadian clockwork is established by an autoregulatory transcriptional-translational feedback loop (TTFL), a molecular mechanism that enables activating and inhibitory components of the clockwork for proper function. Protein dimers of the *Brain and muscle arnt-like 1* (*Bmal1*) and *Circadian locomotor output cycles kaput* (*Clock*) genes form the positive arm of the loop by activating transcription of *Period* (*Per*) and *Cryptochrome* (*Cry*) genes. By their inhibitory interaction with BMAL1/CLOCK, dimerized PER/CRY proteins repress their own transcription, thus forming the negative arm of the loop that repeats every 24 h (Takahashi, 2017). Rhythmic output of the clock is generated by the expression of so-called clock-controlled genes (CCGs) which are involved in the regulation of vital cellular functions like metabolism and cell signaling (Bozek et al., 2009).

Studies have also demonstrated that circadian clock genes play an integral role in the regulation of behavior. Various brain regions, such as the striatum, have been targeted over the past years to elaborate the molecular and neurophysiological mechanism driving the temporal coordination and execution of behaviors related to mood, reward and motor control (Landgraf et al., 2016b; Ozburn et al., 2017; Porcu et al., 2020). The striatum is critically involved in mediating cortical and thalamic input of the basal ganglia, a major network controlling motor, limbic, and associative functions. Two major subregions of the striatum, the caudate putamen (CP) and the nucleus accumbens (NAc), receive dense dopaminergic projections from the substantia nigra (SN) and the ventral tegmental area (VTA), respectively, which is believed to be a key feature in basal ganglia function. Although all GABAergic medium spiny neurons (MSNs), the principal neurons of the striatum, are inhibitory, they can be divided into two distinct subpopulations based on anatomical projections as well as neurochemical and functional properties (Gerfen and Surmeier, 2011). Dopamine receptor type 1 (DRD1) MSNs project directly to the output nuclei and are associated with stimulatory or “go” actions, whereas dopamine receptor type 2 (DRD2) MSNs project indirectly to the striatal output nuclei and are associated with inhibitory “no-go” actions. Even though this classical separation of the two pathways in their distinct roles has been challenged over the past years (Coffey et al., 2017), it has been generally accepted that the direct and indirect pathways provide antagonistic feedback to their output targets.

Dysfunction of striatal dopamine signaling has been linked to the pathophysiology of neurological and psychiatric disorders and circadian clock gene expression appears to play a pivotal role in this context. Besides the rhythmic expression of circadian clock genes and proteins (Feillet et al., 2006; Landgraf et al., 2016b) and daily oscillations of dopamine levels in the striatum (Hood et al., 2010), it has been shown that dopamine signaling, and circadian clock gene expression mutually influence each other. Application of a DRD1 agonist in cultured MSNs evoked stimulatory responses on clock gene expression, whereas treatment with a DRD2 agonist had an inhibitory effect (Imbesi et al., 2009). Interestingly, extracellular dopamine induces the expression of PER2 via the DRD2 pathway, suggesting a crucial function of dopamine in the striatal circadian clockwork (Hood et al., 2010). Vice versa, studies have demonstrated that parts of the dopamine signaling pathway in the striatum are clock-controlled (Ikeda et al., 2013).

Given the comorbidity of circadian rhythm disruption and neuropsychiatric conditions like depression, anxiety, substance abuse, and neurodegenerative disease (Ketchesin et al., 2020), it is tempting to speculate that a disruption of the striatal circadian clock may play a causal role, potentially by its interaction with components of the dopamine signaling pathway (Kim et al., 2017). We tested this hypothesis by assessing behaviors associated with anxiety, depression, and motor function, as well as molecular and physiological properties of MSNs in mice with a selective knockout of *Bmal1* and *Per2* from striatal MSNs.

## Materials and Methods

### Ethics Statement

All experimental protocols and procedures were approved by the Canadian Council on Animal Care and the Concordia University Animal Care Committee (certificate number: 30000256).

### Animals

The conditional ablation of *Bmal1* and *Per2* in striatal MSNs was achieved as described before (de Zavalia et al., 2021). In brief, mice carrying homozygous floxed alleles of the *Bmal1* (*Bmal1* ^*fl/fl*^; B6.129S4(Cg)-Arntl^tm1Weit^/J; stock #: 7668, The Jackson Laboratory) (Storch et al., 2007) or *Per2* locus (*Per2* ^*fl/fl*^; B6.129-Per2^tm1.2Ual^/Biat, strain ID: EM10599, European Mouse Mutant Archive) (Chavan et al., 2016) were crossed with mice expressing Cre recombinase and enhanced green fluorescence protein (EGFP) under control of the *Gpr88* promoter (*Gpr88* ^*cre/+;*^ B6.129S4-Gpr88^tm1.1(cre/GFP)Rpa^/J; stock #: 22,510, The Jackson Laboratory) (Beutler et al., 2011). Gpr88 is an orphan G protein-coupled receptor (GPCR) which is almost exclusively expressed in striatal MSNs (Massart et al., 2009; Quintana et al., 2012). Therefore, the conditional clock gene knockout is restricted to this population of neurons only. Male mice of the F1 generation (*Gpr88* ^*cre/+*^; *Bmal1* ^*fl/+*^, *Gpr88* ^*cre/+*^; *Per2* ^*fl/+*^) were crossed with *Bmal1* ^*fl/fl*^ and Per2^*fl/fl*^ females a second time, respectively, to generate a conditional knockout of *Bmal1* (*Bmal1* cKO) or *Per2* (*Per2* cKO) and corresponding littermate controls (*Bmal1* CTRL, *Per2* CTRL). Because of recombination events at the *Bmal1* and *Per2* locus caused by Cre during germ cell development, the resulting experimental and control animals had only one functional copy of the respective target gene genome-wide. The genotypes were as following: *Bmal1* conditional knockout (cKO, *Gpr88* ^*cre/+*^; *Bmal1* ^*-/fl*^), *Bmal1* controls (CTRL, *Gpr88* ^*+/+*^; *Bmal1* ^*-/fl*^), *Per2* cKO (*Gpr88* ^*cre/+*^; *Per2* ^*-/fl*^), *Per2* CTRL (*Gpr88* ^*+/+*^; *Per2* ^*-/fl*^). To rule out that behavioral and physiological alterations in *Bmal1* and *Per2* cKO animals were a result of a *Gpr88* haploinsufficiency, male and female *Gpr88* ^*cre/+*^ and *Gpr88* ^*+/+*^ controls were assessed as well.

The *Bmal1*, *Per2* and *Gpr88* transgenic mouse lines were backcrossed for at least six generations onto a C57BL/6J background. DNA for genotyping was obtained from tail biopsies and analyzed using a standard PCR-based protocol (M0484L, New England Biolabs Inc., Ipswich, MA, United States) with the following primers: *Gpr88*: F 5′-AAG​AAC​CTG​ATG​GAC​ATG​TT-3′ and R 5′-CCA​CCG​TCA​GTA​CGT​GAG​AT-3′ resulting in a 500 bp product in mutant mice; *Bmal1*: F1 5′-ACT​GGA​AGT​AAC​TTT​ATC​AAA​CTG-3′, F2 5′-CTC​CTA​ACT​TGG​TTT​TTG​TCT​GT-3′, R 5′-CTG​ACC​AAC​TTG​CTA​ACA​ATT​A-3′ resulting in products of 327 bp for wild type, 431 bp product for floxed and a ∼580 bp product for Bmal1-ablated alleles; *Per2*: F 5′-AGC​TGG​CAA​AGG​TCA​CTC-3′, R 5′- GCA​GGG​CAG​TTT​CAT​CAA​GG-3′ resulting in products of 1,013 bp for floxed, 721 for wild type and 450 bp for *Per2* knockout alleles.

Animals were group-housed (two to four animals per cage) and kept under a 12:12 h light-dark (LD) cycle, with food and water available *ad libitum* unless stated otherwise. Room temperature and relative humidity varied around 22 ± 2°C and 65 ± 5%, respectively. The bedding of the cages was changed every week.

### Behavioral Procedures

Naïve, 12–16 weeks old animals were exposed to a series of behavioral tests to assess anxiety- and depressive-like behaviors and motor functions. The order of the procedures was fixed, starting with the elevated plus maze test (EPM) followed by the open field test (OFT), marble burying test (MBT), horizontal bar test (HBT), fixed speed rotarod (FSRR), tail suspension test (TST) and dopamine receptor agonist administration. The interval between experiments was at least 1 week. Experiments were carried out in a separate experimental room. The room was moderately lit during the light phase (500 lux), whereas procedures were conducted under dim red light (∼2 lux) during the dark phase. Before each test, animals were habituated to the test room for 1 h.

### Elevated Plus-Maze Test

Anxiety-related behavior was assessed in an elevated plus-maze test. The cross-shaped maze (Panlab, Barcelona, Spain) was elevated 40 cm above the floor and consisted of two open and closed arms (6 cm × 29.5 cm each) and a central area (6 cm × 6 cm). The maze was equipped with white floors and black walls (15 cm in height) in the closed arm made of Methacrylate. Animals were randomly assigned into two groups and tested either between Zeitgeber Time (ZT) 2–6 (ZT0 represents the time of light onset) or ZT14–18.

Animals were gently transferred into the central area of the maze facing the closed arm and video-recorded for 5 min using a Samsung Galaxy A5 2017 phone. The recording device was mounted right above the center of the EPM. Animals were weighed and transferred back to their home cage after the test was completed. The EPM was thoroughly wiped with 70% ethanol solution after each trial.

Videos files were converted into 1-s image sequences using VirtualDub1.10.4 and further compiled to multi-Tiff files using FIJI (Schindelin et al., 2012). Multi-Tiff files were analyzed by the ImageEP (Ver. 1,201,112) plugin in FIJI (Komada et al., 2008) to determine distance travelled, time spent in the open arms of the EPM, as well as the number of entries into the open arms.

### Open Field Test

The open field test has been conducted to assess anxiety-related behavior and motor activity using an automated tracking system (Panlab, Barcelona, Spain). The same groups of mice as described for the EPM were tested at ZT2–6 or ZT14–18. The system was composed of two frames equipped with infrared beams for subject detection in a Plexiglas arena (44 cm × 44 cm × 30 cm). Mice were gently introduced into the corner of the open field facing the wall and spontaneous horizontal and vertical activity was recorded for each animal individually over a 60 min period by the Actitrack software (Panlab, Barcelona, Spain). At the end of the session, animals were weighed and returned to their home cages. The arena was wiped with 70% ethanol to remove any residues and olfactory cues thereafter.

The Actitrack software was also used to analyze the tracking data. Total distance travelled, permanence time in the central area of the open field, and rearing behavior was assessed.

### Marble Burying Test

The marble burying test has been used to study repetitive and anxiety-related behavior. Standard plastic cages (43 cm × 21 cm × 21 cm) were filled with standard bedding (5 cm) and 20 multi-colored glass marbles (16 mm diameter, Editions Gladius International, Quebec, QC, Canada) were laid out equidistantly in a 5 × 4 grid. Mice were gently transferred into a corner of the cage at ZT2 and left in the cage for 30 min. Mice were weighed and transferred back to their home cages at the end of the test, and the number of buried marbles was counted thereafter. A marble was considered buried when 2/3 of its surface was covered in the bedding, and the total number of buried marbles was used to calculate the percentage of buried marbles for each subject.

### Horizontal bar Test

Motor coordination was assessed in a horizontal bar test between ZT6–8. A metal bar (ø 2.5 and 5 mm) was mounted between two opposite sides of a squared cardboard box (41 cm × 41 cm × 41 cm), and the bottom of the box was covered with a soft pad to cushion animals falling from the bar.

During the first stage of the experiment, mice were gently held by the tail and lifted towards the horizontal bar (ø 2.5 mm) where they had to grasp the bar at the central point with the forepaws before being released. The criterion within a 60 s trial period was either reaching one end of the bar or falling from the bar before reaching the end of it. The test was repeated three times with 30 s resting periods between each trial. Stage two of the HBT followed the same experimental design immediately after the first stage, except that the thicker bar (ø 5 mm) was used.

Each trial was video recorded for later analysis. A score was calculated for each trial based on the mice’s performance. For reaching the end of the bar, the score was calculated by subtracting the time reaching the end of the bar from 120 (score pass = 120–time pass). The score for falling represented the same time it took the mice to fall from the bar (score fall = time fall). If a mouse was staying on the bar during the entire trial period without reaching the end, a score of 60 was given (score stay = 60). A cumulative score from the averages of the three trials of each experimental stage was calculated.

### Rotarod

A custom-made, fixed-speed rotarod (FSRR) was used to further assess motor coordination in experimental and control mice of both sexes between ZT6–8. In brief, the rotarod was composed of a rod (ø 5 cm) mounted 19.5 cm above the base of the device. The base was separated into 6 individual compartments equipped with a soft foam pad to cushion mice falling off the rod. To prevent mice from leaving the rod, two flanges (ø 20 cm) separated a 9.3 cm interspace on the rod. The rod was driven by an electric motor, and a gearbox was used to set the desired speed.

On the day of the experiment, animals underwent a training session on the rotarod between ZT2–4 to familiarize themselves with the apparatus. For this, animals were gently grabbed by their tail and carefully placed on the rod turning at 2 rpm for 5 min. Animals falling from the rod were always put back on the rod throughout the entire training period. After the training, animals were transferred back to their home cages. Mice underwent testing between ZT6–8 the same day. While being video-recorded, animals were positioned on the turning rod as described above and left on the rod for a 1-min trial period, except that they were not put back on the rod when falling from it within the trial. If a mouse fell off the rod due to poor placement by the experimenter, the trial was not counted and repeated after a short resting time. Mice were gently transferred into the compartment at the base of the Rotarod at the end of each trial and left for a 30 s resting period. Each trial was repeated two more times before the rod was set to a higher speed. The starting speed was 2 rounds per minute (rpm), which was gradually increased to 4, 8 and 12 rpm. If animals failed to stay on the rod for the 1-min period at all 3 trials, they were not further tested at higher speeds.

The time staying on the rod was measured using a stopwatch. The average time on the rod calculated from the three replicates of each speed level was calculated and summed up for a final score.

### Tail Suspension Test

Depressive-like behavior was evaluated in the tail suspension test at ZT8. Animals were suspended by their tails above the ground for 6 min while being video recorded. Climbstoppers were placed over the tail to prevent mice from tail climbing (Can et al., 2012). After the test, animals were weighed and returned to their home cages. Two experienced researchers blinded to the animals’ genotypes analyzed the video recordings using an on-screen stopwatch (Stopwatch+, Center for Behavioral Neuroscience, Atlanta, GA, United States). The total amount of mobility time, defined as any movement of the body, was scored. Immobility time was calculated by subtracting mobility time from the total duration of the experiment.

### Dopamine Receptor Agonist Administration

The effect of systemic dopamine receptor agonist administration on locomotor behavior was assessed in male and female *Bmal1* and CTRL mice, as well as male and female *Gpr88* ^*cre/+*^ and *Gpr88* ^*+/+*^ animals. For this, animals were randomly assigned into two groups (saline control group vs. dopamine receptor agonist-treated group) and baseline locomotor activity levels were recorded in the open field (Panlab, Barcelona, Spain) for 30 min at ZT2. Immediately after baseline activity recording, animals received intraperitoneal injections (10 ml/kg body weight) of saline or the DRD1 agonist SKF-81297 (5 mg/kg body weight, Cayman Chemical, Ann Arbor, MI, United States) and locomotor activity was recorded for another 60 min in the open field. The procedure was repeated after 6–10 weeks, but DRD2 agonist quinpirole (1 mg/kg body weight, Sigma-Aldrich, St. Louis, MO, United States) was administered instead.

The total distance travelled within the 60 min post-injection period was analyzed relative to baseline activity.

### Mitochondrial Respiration

A sequential substrate addition protocol was conducted to assess mitochondrial coupled and uncoupled oxygen consumption, leak and membrane integrity, using a polarographic sensor (Oxygraph-2k, Oroboros Instruments, Innsbruck, Austria). The measurements of oxygen consumption were performed in MiR05 at 37°C. MiR05 contains (in mM) EGTA 0.5, MgCl_2_·6H_2_O 3.0, K-lactobionate 60, taurine 20, KH_2_PO_4_ 10, HEPES 20, sucrose 110, BSA 1 g/L, and pH 7.1. The oxygen flux was registered and analyzed by the DatLab 7.0 software.

Between 2 and 3 mg (wet weight) of fresh striatal tissue punches were collected at ZT2 and ZT6 from male and female *Bmal1* cKO and CTRL animals as well as *Gpr88* ^*cre/+*^ and *Gpr88* ^*+/+*^ mice (20–27 weeks old). The tissue was placed in the Oxygraph after which oxygen levels were increased to approximately 480 pmol. 50 μg/ml of Saponin was then added to the chambers to permeabilize the tissue before the actual experiment started.

In the protocol, non-phosphorylating LEAK-respiration was induced by adding the complex I (CI)-linked substrates malate (2 mM), pyruvate (5 mM), and glutamate (5 mM). Subsequently, oxidative phosphorylation (OXPHOS) capacity of CI-linked activity was measured after addition of a saturating concentration of ADP (5 mM). Next, Cytochrome c (10 μM) was added to assess for mitochondrial membrane damage. OXPHOS-capacity with combined CI and II-linked substrates was assessed by addition of succinate (10 mM). This was followed by FCCP [carbonylcyanide-4-(trifluoromethoxy)phenylhydrazone, 1 μM] to test uncoupling and then Antimycin A (2.5 μM) which was used for blocking complex II (CIII) to investigate non-mitochondrial oxygen consumption.

The degree of coupling between oxidation and phosphorylation expressed as respiratory acceptor control ratio (ACR) was calculated by the rate of oxygen consumption while ADP is being phosphorylated divided by the rate of non-phosphorylating LEAK-respiration induced by glutamate administration.

### Gene Expression

Gene expression was measured in tissue punches collected from the caudate putamen (CP) at ZT2, 8, 14 and 20. Brains from decapitated *Bmal1* cKO and CTRL male and female mice (18–25 weeks old) were flash-frozen after extraction and stored at −80°C until further processing. 100–150 μm thick coronal sections were obtained from a cryostat (Microm HM 505 E, Microm International GmbH, Walldorf, Germany), and tissue punches of the CP (1.5 mm in diameter) were collected from each hemisphere in accordance with the mouse brain atlas (Paxinos and Franklin, 2004).

A standard Trizol extraction protocol was used for total RNA isolation according to the manufacturer’s instructions (Invitrogen™, Life Technologies Corporation, Carlsbad, CA, United States). Total RNA yield was measured using spectrophotometry (Nanodrop 2000; Thermo Scientific™, Wilmington, DE, United States), and RNA integrity was assessed by the “bleach gel” method (Aranda et al., 2012). cDNA was synthesized from 1 μg of RNA using the iScriptTM Reverse Transcription Supermix following the manufacturer’s instructions (Biorad, Hercules, CA, United States). Besides the standard cDNA samples, a no reverse transcriptase (no-RT) control was prepared.

SYBR green-based quantitative real-time PCR was used to analyze gene expression. Triplicates of 10 μL reactions containing SsoAdvanced Universal SYBR^®^ Green Supermix (Biorad, Hercules, CA, United States) and 300 μM of the respective forward and reverse primers were amplified using a CFX96TM Real-Time PCR system (Biorad, Hercules, CA, United States). The list of forward and reverse primers is provided in Table 1.

**TABLE 1:** Primer list for quantitative real-time PCR.

Target gene	Forward primer	Reverse primer	Efficiency (%)
Gapdh	CAT​CTT​CCA​GGA​GCG​AGA​CC	GGC​GGA​GAT​GAT​GAC​CCT​TT	100.9
Ppib	GGA​GAT​GGC​ACA​GGA​GGA​AA	CCG​TAG​TGC​TTC​AGC​TTG​AAG​TTC​T	100.9
Bmal1	GGA​CAC​AGA​CAA​AGA​TGA​CCC​T	ATT​TTG​TCC​CGA​CGC​CTC​TT	107.7
Per1	CCT​TCC​TCC​ACA​CCG​ACA​AA	GCT​GCA​TGG​CTC​CTA​ACT​GA	102.9
Per2	ACA​GAA​GGA​AGA​GCA​AGC​CT	GCT​TTA​GAT​CGG​CAG​TGG​TG	110.9
Cry1	GAG​CAG​GTT​GAG​GGC​ATT​AC	AGG​CGT​CCT​TCT​TAC​AGT​GA	96.6
Dbp	TGT​CAA​GCA​TTC​CAG​GCC​AT	TCC​GGC​TCC​AGT​ACT​TCT​CA	106.5
Drd1	CTG​CTG​GCT​CCC​TTT​CTT​CA	GGG​GTT​CAG​GGA​GGA​ATT​CG	101.2
Drd2	CAT​TGT​CTG​GGT​CCT​GTC​CT	GAA​CGA​GAC​GAT​GGA​GGA​GT	99.3
Maoa	TGG​AGT​GGC​TAC​ATG​GAA​GG	CCT​TGG​ACT​CAG​GCT​CTT​GA	101.7
Gad67	GGA​GCT​GGC​TGA​TTA​CCT​CT	GCT​ATC​TGG​AAC​CCC​TCG​AA	97.4
Sirt1	TCG​TGG​AGA​CAT​TTT​TAA​TCA​GG	GCT​TCA​TGA​TGG​CAA​GTG​G	100.8

The CFX Maestro qPCR Software (Biorad, Hercules, CA, United States) and Microsoft Excel were used to calculate relative normalized changes in gene expression by the delta-delta Ct (∆∆Ct) method (Pfaffl, 2001). For this, expression levels of target genes relative to two reference genes were further normalized CTRL males at ZT2.

### Data Analysis and Statistics

Data from each behavioral test, mitochondrial respiration measurements as well as gene expression analysis were further processed and analyzed using Prism 9 (GraphPad Software, San Diego, CA, United States). Results were depicted as mean ± standard error of the mean (SEM). Behavioral tests conducted at two different time points were analyzed using a three-way analysis of variance (ANOVA, genotype, sex, time point effect) followed by Šídák’s multiple comparisons if statistically significant main effects were found. Behavioral tests conducted at one time point were analyzed by a two-way ANOVA (genotype and sex effect) followed by Tukey’s multiple comparisons. Interval data of locomotor activity recorded in the open field were analyzed using a three-way repeated-measures ANOVA (genotype, treatment, and interval effect) followed by Bonferroni’s multiple comparisons test. Measurements of mitochondrial respiration in mice of the *Bmal1* line were analyzed using a three-way ANOVA (treatment, genotype, and time point effect) followed by Šídák’s multiple comparisons, whereas data from the Gpr88 line were analyzed using a two-way ANOVA, which was subsequently compared by a by Šídák’s multiple comparisons if significant main effects were found. A *t*-test was conducted for between-group comparisons of the ACR. A three-way ANOVA (genotype, sex, and time point effect) followed by Tukey’s multiple comparisons was used to assess results from the gene expression analysis. A cosine analysis and subsequent zero-amplitude test, which is based on F-statistics, were conducted to further assess the significance of daily oscillations in gene expression (Cornelissen, 2014). The level of significance was set at *p* ≤ 0.05.

## Results

### Anxiety- and Depressive-Like Behavior

**FIGURE 1:**
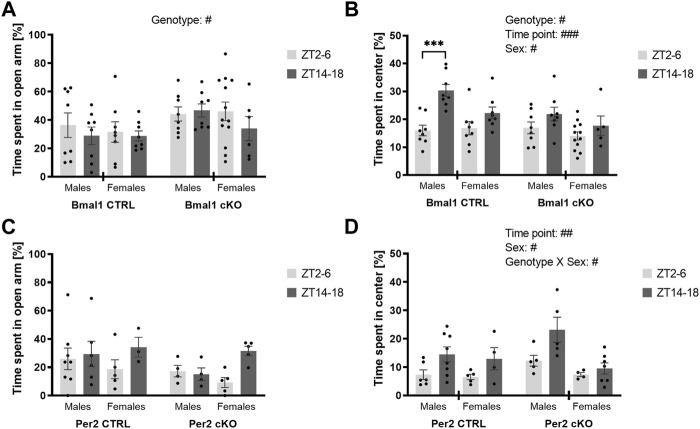
Anxiety-like behavior in mice with a striatal-specific clock gene knockout. Time spent in the open arm of the elevated plus-maze and time spent in the center of an open field was investigated during the light (ZT2–6, light grey bar) and the dark period (ZT 14–18, dark grey bar). Mice with a conditional *Bmal1* knockout (n = 6–13 per genotype, sex, and time point) displayed decreased anxiety-like behavior in the elevated plus-maze compared to controls **(A)**, whereas increased anxiety-like behavior was observed in the open field test **(B)**. The conditional knockout of *Per2* (n = 3–8 per genotype, sex, and time point) did not affect anxiety-like behavior in the elevated plus-maze and open field **(C,D)**. Results are depicted as mean ± standard error of the mean (S.E.M.). # … *p* ≤ 0.05, ## … *p* ≤ 0.05, ### … *p* ≤ 0.05, three-way ANOVA. *** … *p* ≤ 0.0005, Šídák’s multiple comparisons test. Details of the statistical analysis are shown in Table 2.

**FIGURE 2:**
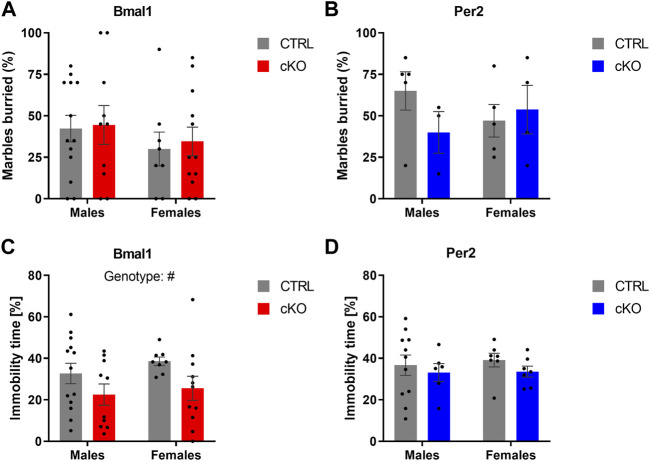
Anxiety- and depressive-like behavior in mice with a conditional *Bmal1* or *Per2* knockout and control animals. No genotype effects on the number of buried marbles were found in male and female mice of the two mouse lines (n = 3–13 per genotype, sex, and mouse line) **(A,B)**. Depressive-like behavior indicated by immobility time in the tail suspension test is decreased in mice with a striatal *Bmal1* knockout (n = 8–13 per genotype and sex) **(C)** but unaffected in mice with a conditional ablation of *Per2* (n = 6–11 per genotype and sex) from striatal MSNs **(D)**. Results are depicted as mean ± standard error of the mean (S.E.M.). # … *p* ≤ 0.05, three-way ANOVA. Details of the statistical analysis are shown in Table 2.

The conditional ablation of *Bmal1* or *Per2* from striatal MSNs affected anxiety-related behaviors differently. Details of the statistical analysis are depicted in Table 2. Mice with a conditional knockout of *Bmal1* spent more time in the open arm of the elevated plus-maze compared to controls (Figure 1A). The results were supported by a higher number of open arm entries of cKO mice (Supplementary Figure 1B), whereas general levels of activity in the EPM were not affected by genotype (Supplementary Figure 1A). Subsequent assessment of mice in the open field test further emphasized the impact of a conditional knockout of *Bmal1* on anxiety-related behaviors, with opposite effects as observed in the EPM test. *Bmal1* cKO animals spent less time in the central area of the open field compared to CTRL mice (Figure 1B). In contrast, no effect of the striatal-specific *Bmal1* deletion was observed in the marble-burying task (Figure 2A).

**TABLE 2:** Statistical analysis of affective behaviours and motor function.

Assay	Mouse line	Three-way ANOVA factors			
		Genotype	Time point	Sex	Interaction
EPM open arm time	*Bmal1*	F _(1, 59)_ = 5.784, *p* = 0.0193	n.s.	n.s.	n.s.
	*Per2*	n.s.	n.s.	n.s.	n.s.
	*Gpr88*	n.s.	F _(1, 54)_ = 9.463, *p* = 0.0033	n.s.	n.s.
EPM open arm entries	*Bmal1*	F _(1, 59)_ = 7.553, *p* = 0.0079	n.s.	n.s.	n.s.
	*Per2*	F _(1, 32)_ = 4.174, *p* = 0.0494	F _(1, 32)_ = 7.399, *p* = 0.0105	n.s.	n.s.
	*Gpr88*	n.s.	F _(1, 54)_ = 8.104, *p* = 0.0062	n.s.	n.s.
EPM distance travelled	*Bmal1*	n.s.	F _(1, 59)_ = 6.395, *p* = 0.0141	F _(1, 59)_ = 4.390, *p* = 0.0405	n.s.
	*Per2*	n.s.	n.s.	n.s.	n.s.
	*Gpr88*	n.s.	F _(1, 54)_ = 15.92, *p* = 0.0002	n.s.	n.s.
OFT center time	*Bmal1*	F _(1, 57)_ = 5.802, *p* = 0.0193	F _(1, 57)_ = 20.86, *p* < 0.0001	F _(1, 57)_ = 5.275, *p* = 0.0253	n.s.
	*Per2*	n.s.	F _(1, 36)_ = 12.76, *p* = 0.0010	F _(1, 36)_ = 7.836, *p* = 0.0082	genotype X sex, F _(1, 36)_ = 4.677, *p* = 0.0373
	*Gpr88*	n.s.	F _(1, 54)_ = 23.36, *p* < 0.0001	n.s.	n.s.
OFT distance travelled	*Bmal1*	F _(1, 57)_ = 25.96, *p* < 0.0001	F _(1, 57)_ = 6.718, *p* = 0.0121	F _(1, 57)_ = 5.747, *p* = 0.0198	n.s.
	*Per2*	F _(1, 36)_ = 19.50, *p* < 0.0001	n.s.	n.s.	n.s.
	*Gpr88*	F _(1, 54)_ = 16.37, *p* = 0.0002	F _(1, 54)_ = 13.99, *p* = 0.0004	n.s.	n.s.
OFT rearing	*Bmal1*	F _(1, 57)_ = 12.35, *p* = 0.0009	F _(1, 57)_ = 19.59, *p* < 0.0001	n.s.	n.s.
	*Per2*	n.s.	n.s.	n.s.	n.s.
	*Gpr88*	F _(1, 54)_ = 5.915, *p* = 0.0184	F _(1, 54)_ = 15.65, *p* = 0.0002	n.s.	n.s.
MBT # marbles buried	*Bmal1*	n.s.	n.s.	n.s.
	*Per2*	n.s.		n.s.	n.s.
	*Gpr88*	n.s.		F _(1, 45)_ = 13.95, *p* = 0.0005	n.s.
TST immobility time	*Bmal1*	F _(1, 38)_ = 5.146, *p* = 0.0291		n.s.	n.s.
	*Per2*	n.s.		n.s.	n.s.
	*Gpr88*	n.s.		n.s.	n.s.
HBT bar performance	*Bmal1*	F _(1, 38)_ = 5.206, *p* = 0.0282		n.s.	n.s.
	*Per2*	n.s.		n.s.	n.s.
	*Gpr88*	n.s.		n.s.	n.s.
FSSR time on rod	*Bmal1*	F _(1, 38)_ = 15.67, *p* = 0.0003		n.s.	n.s.
	*Per2*	n.s.		n.s.	n.s.
	*Gpr88*	n.s.		n.s.	n.s.

Whereas a striatal-specific knockout of *Per2* did not affect the time spent in the open arm of the elevated plus-maze, the number of open arm entries was reduced in *Per2*-cKO mice (Figure 1C, Supplementary Figure 1F). However, the level of activity displayed in the EPM was similar between knockout animals and controls (Supplementary Figure 1E). Further assessment of anxiety-like behavior in the open-field and marble-burying test revealed no effect of the conditional *Per2* knockout (Figures 1D, 2B).

Depressive-like behavior was affected by the conditional knockout of *Bmal1* but not *Per2*. A two-way ANOVA revealed a significant effect of genotype (Figure 2C), indicating that *Bmal1* cKO mice displayed lower immobility time while being suspended on the bar in the tail suspension test. Immobility time was not different between *Per2*-cKO and CTRL mice (Figure 2D).

Importantly, the loss of one copy of *Gpr88* did not affect anxiety- or depressive-like behaviors displayed in the elevated plus-maze test (Supplementary Figures 2A,C), open field test (Supplementary Figure 2D), marble-burying test (Supplementary Figure 3A) or tail suspension test (Supplementary Figure 3B) (Table 2). Time of day, however, was a determining factor on behavior displayed in the elevated plus-maze and open field in mice of the *Gpr88* strain. The results indicate diurnal differences in anxiety-like behavior displayed in the elevated-plus maze and open field, but with opposite direction depending on the test. Diurnal differences were present in some of the behaviors displayed in the elevated plus-maze and open field in mice of the *Bmal1* and *Per2* line as well, but less pronounced compared to mice of the *Gpr88* line. However, the results of the statistical analysis do not demonstrate that the effect is due to the conditional knockout of *Bmal1* or *Per2* because significant interactions between genotype and time point were not found (Table 2).

### Motor Function and Coordination

Mice with a conditional knockout of *Bmal1* and *Per2* showed increased levels of locomotor activity in the open field compared to CTRL animals. Genotype effects were found for both, total distance travelled and rearing activity in animals of the *Bmal1* line (Supplementary Figures 1C,D), whereas total distance travelled but not rearing activity was increased in *Per2* cKO mice (Supplementary Figures 1G,H) (Table 2).

Also, mice of the *Gpr88* ^*(cre/+)*^ genotype displayed increased levels of locomotor activity in the open field compared to their *Gpr88* ^*(+/+)*^ counterparts and had higher levels of rearing activity (Supplementary Figures 2E,F).

Besides genotype, the time point of testing and sex were factors affecting activity levels in the open field. In mice of the *Bmal1* line, spikes in total distance travelled varied depending on sex and time of day (Supplementary Figure 1C). Also, rearing activity was more pronounced during the dark period (Supplementary Figure 1D). Horizontal and vertical activity was not affected by time of testing and sex in mice of the *Per2* line. Animals of the *Gpr88* line displayed a higher level of locomotion in the open field during the dark phase. A time point effect was found in both, total distance travelled and rearing activity (Table 2).

**FIGURE 3:**
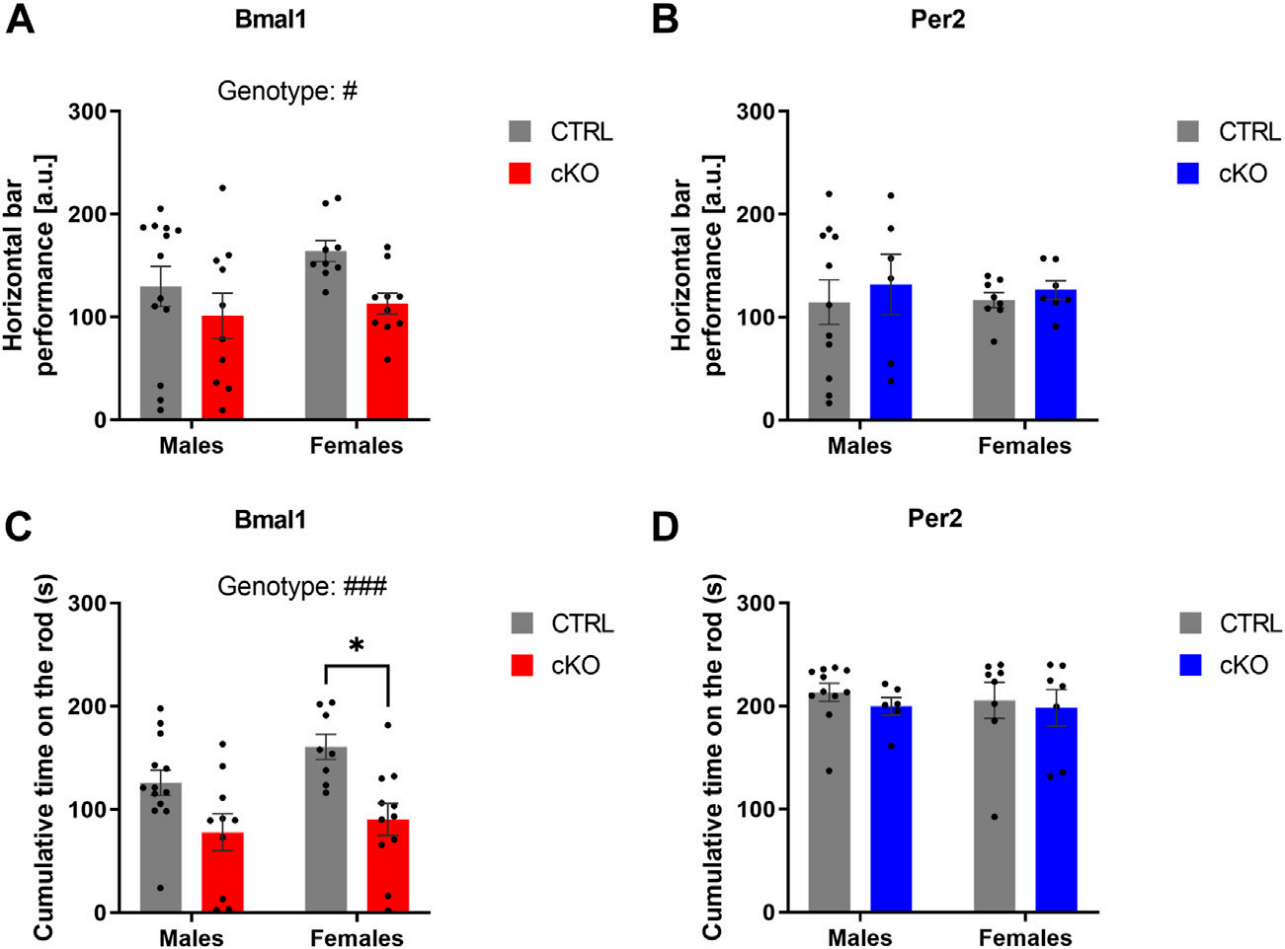
Motor functions in mice with a conditional *Bmal1* or *Per2* knockout and controls. Mice with a striatal-specific *Bmal1* knockout (n = 9–13 per genotype and sex) performed worse in the horizontal bar test compared to controls **(A)**, whereas no differences were found in mice with a knockout of *Per2* (n = 6–11 per genotype and sex) **(B)** from striatal MSNs. Likewise, *Bmal1* knockout mice (n = 8–13 per genotype and sex) **(C)** had deficits in motor coordination assessed in the rotarod in contrast to mice with a conditional ablation of *Per2* (n = 6–11 per genotype and sex) **(D)** from striatal MSNs. Results are depicted as mean ± standard error of the mean (S.E.M.). # … *p* ≤ 0.05, ### … *p* ≤ 0.0005, three-way ANOVA. * … *p* ≤ 0.05, Tukey’s multiple comparisons test. Details of the statistical analysis are shown in Table 2.

Motor coordination was assessed in the horizontal bar and rotarod test in males and females of all three mouse lines. Whereas a conditional ablation of *Per2* or the deletion of on copy of *Gpr88* from striatal MSNs did not affect the performance on the horizontal bar test, a genotype effect was revealed in animals of the *Bmal1* line, indicating a poorer performance in *Bmal1* cKO animals (Figure 3A). Deficits in motor coordination in *Bmal1* cKO mice were furthermore confirmed in the rotarod test (Figure 3C), whereas no difference was found in mice of the *Per2* and *Gpr88* lines. Details of the statistical analysis are depicted in Table 2.

### Response to Dopamine Receptor Agonist Administration

**FIGURE 4:**
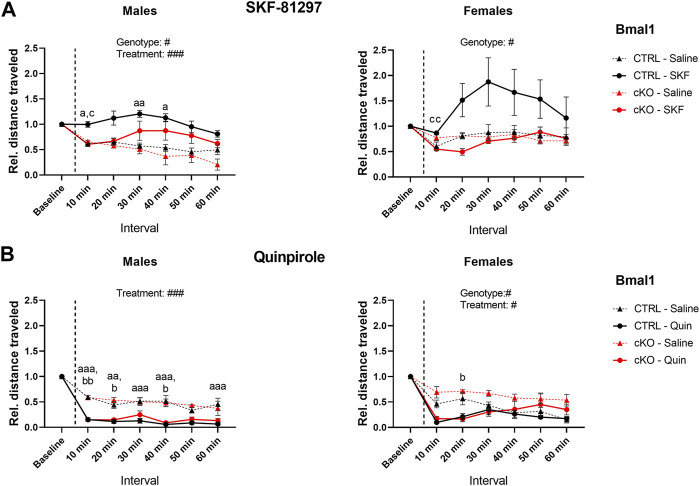
Locomotor response to DRD1 and DRD2 agonist administration in mice with a conditional *Bmal1* knockout. Changes in activity relative to baseline levels are depicted in 10-min intervals. Males and females with a conditional knockout of *Bmal1* (n = t3–5 per sex and treatment) displayed an attenuated response to SKF-81297 compared to controls (n = 4–7 per sex and treatment) **(A)**. Activity was inhibited in males and females with a conditional knockout of *Bmal1* (n = 3–4 per sex and treatment) and controls (n = 4–8 per sex and treatment) following Quinpirole administration compared to saline injections **(B)**. Results are depicted as mean ± standard error of the mean (S.E.M.). Only genotype and treatment main effects are displayed in the graph. # … *p* ≤ 0.05, ### … *p* ≤ 0.0005, three-way ANOVA. Letters indicate levels of statistical difference between genotype and treatment post-hoc comparison: a … treatment effect CTRL group, b … treatment effect cKO group, c … genotype effect for SKF or Quinpirole treatment; one letter … *p* ≤ 0.05, two letters … *p* ≤ 0.005, three letters … *p* ≤ 0.0005, Tukey’s multiple comparisons test. Details of the statistical analysis are displayed in Table 3.

Administration of dopamine receptor agonists evoked different responses depending on the type of agonist and genotype. Although locomotor activity was induced by DRD1 receptor agonist injections in *Bmal1* cKO and CTRL males compared to saline controls, the effect of the treatment appeared to be delayed in cKO animals when compared to CTRL mice. In females, a significant main effect of genotype but no treatment effect was found. Nevertheless, the results indicate a stronger response of CTRL mice to SKF treatment compared to cKO animals (Figure 4A).

In males of the *Gpr88* line, a similar trend was observed compared to the findings in males of the *Bmal1* line. Both, *Gpr88* ^*(cre/+)*^ and *Gpr88* ^*(+/+)*^ animals displayed increased activity following the SKF injection compared to saline controls, which appeared to be delayed in *Gpr88* ^*(cre/+)*^ animals (Supplementary Figure 4A). Females of the *Gpr88* line displayed a similar response to males. Whereas the treatment affected locomotion significantly, no genotype effect was found.

In contrast, administration of the DRD2 agonist Quinpirole had inhibitory effects on locomotor activity in male and female individuals of both mouse lines. *Bmal1* cKO and CTRL males displayed significantly reduced locomotion following the administration of Quinpirole compared to saline control animals. Females showed a similar response, however, the effect seemed to be less pronounced compared to males (Figure 4B). Moreover, activity levels in cKO females tend to be higher compared to CTRL animals irrespective of the treatment.

*Gpr88* ^*(cre/+)*^ and *Gpr88* ^*(+/+)*^ male and female mice displayed reduced locomotor activity following quinpirole administration when compared to saline controls (Supplementary Figure 4B), independently of genotype. Details of statistical analysis are shown in Table 3.

**TABLE 3:** Statistical analysis of dopamine receptor agonist treatment and mitochondrial respiration.

Assay/mouse line	Sex	Three-way ANOVA factors			
		Genotype	Interval	Treatment	Interaction
D1 agonist SKF-81297	Males	F _(1, 18)_ = 8.890, *p* = 0.0080	F _(2.710, 48.78)_ = 14.01, *p* < 0.0001	F _(1, 18)_ = 32.14, *p* < 0.0001	Interval X treatment
					F _(6, 108)_ = 6.509, *p* < 0.0001
*Bmal1*	Females	F _(1, 12)_ = 5.213, *p* = 0.0414	F _(2.272, 27.26)_ = 3.229, *p* = 0.0495	n.s.	Interval X genotype
					F _(6, 72)_ = 2.719, *p* = 0.0195
D2 agonist Quinpirole	Males	n.s.	F _(3.106, 55.92)_ = 104.7, *p* < 0.0001	F _(1, 18)_ = 88.74, *p* < 0.0001	Interval X treatment
					F _(6, 108)_ = 8.006, *p* < 0.0001
*Bmal1*	Females	F _(1, 11)_ = 5.461, *p* = 0.0394	F _(3.208, 35.28)_ = 47.70, *p* < 0.0001	F _(1, 11)_ = 10.83, *p* = 0.0072	Interval X treatment
					F _(6, 66)_ = 6.584, *p* < 0.0001
D1 agonist SKF-81297	Males	n.s.	F _(1.918, 26.85)_ = 6.800, *p* = 0.0045	F _(1, 14)_ = 11.37, *p* = 0.0046	Interval X treatment
					F _(6, 84)_ = 3.481, *p* = 0.0040
*Gpr88*	Females	n.s.	F _(2.470, 51.86)_ = 9.164, *p* = 0.0002	F _(1, 21)_ = 6.176, *p* = 0.0215	Interval X treatment
					F _(6, 126)_ = 2.713, *p* = 0.0164
D2 agonist Quinpirole	Males	n.s.	F _(3.727, 55.91)_ = 145.3, *p* < 0.0001	F _(1, 15)_ = 35.34, *p* < 0.0001	Interval X treatment
					F _(6, 90)_ = 9.159, *p* < 0.0001
					Interval X genotype
					F _(6, 84)_ = 3.242, *p* = 0.0065
					Interval X treatment
					F _(6, 84)_ = 6.930, *p* < 0.0001
*Gpr88*	Females	n.s.	F _(3.239, 45.34)_ = 86.79, *p* < 0.0001	F _(1, 14)_ = 86.72, *p* < 0.0001	
Mitochondrial respiration	Males	n.s.	n.s.	F _(7, 64)_ = 176.5, *p* < 0.0001	n.s.
*Bmal1*	Females	n.s.	n.s.	F _(7, 64)_ = 174.8, *p* < 0.0001	n.s.
Mitochondrial respiration	Males	n.s.	n.s.	F _(7, 32)_ = 34.32, *p* < 0.0001	n.s.
*Gpr88*	Females	n.s.	n.s.	F _(7, 32)_ = 17.20, *p* < 0.0001	n.s.

### Striatal Mitochondrial Respiration

**FIGURE 5:**
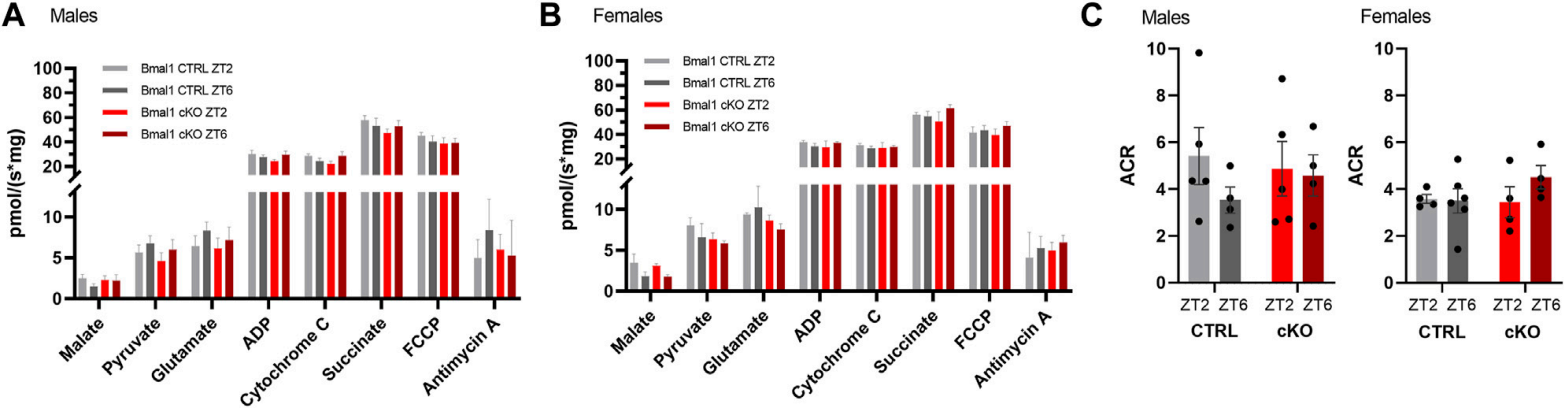
Striatal mitochondrial respiration in mice with a conditional knockout of *Bmal1* and control animals. Sequential substrate addition to assess mitochondrial coupled and uncoupled oxygen consumption, as well as leak and membrane integrity, revealed no differences between male and female mice with a conditional *Bmal1* knockout and controls (n = 4–6 per genotype, sex, and time point) **(A,B)**. Likewise, the degree of coupling between oxidation and phosphorylation did not differ between genotypes within the *Bmal1* mouse line **(C)**. Results are depicted as mean ± standard error of the mean (S.E.M.). Details of the statistical analysis are displayed in Table 3.

High-resolution respirometry in striatal tissue revealed a substantial change in oxygen consumption following a sequential substrate addition to assess complex I respiration, mitochondrial membrane damage, the capacity of oxidative phosphorylation and mitochondrial uncoupling in males and females of the *Bmal1* line. Mitochondrial respiration, however, was unaffected by the conditional knockout of *Bmal1* from striatal MSNs or the time point of measurement (Figures 5A,B). A treatment effect was also found in males and females of the *Gpr88* line irrespective of the genotype (Supplementary Figures 5A,B). Details of the statistical test are shown in Table 3.

Comparison of the degree of coupling between oxidation and phosphorylation as an indirect assessment of mitochondrial efficiency between *Bmal1* cKO and CTRL males and females did not reveal differences between genotypes or time of testing (Figure 5C). Similarly, no differences were observed between *Gpr88* ^*(cre/+)*^ and *Gpr88* ^*(+/+)*^ males or females (Supplementary Figure 5C), even though the ACR was considerably lower in animals of the *Gpr88* line compared to mice of the *Bmal1* line (males gene effect: F_1, 12_ = 5.058, *p* < 0.05; females gene effect: F_1, 10_ = 19.34, *p* < 0.05, two-way ANOVA).

### Gene Expression

**FIGURE 6:**
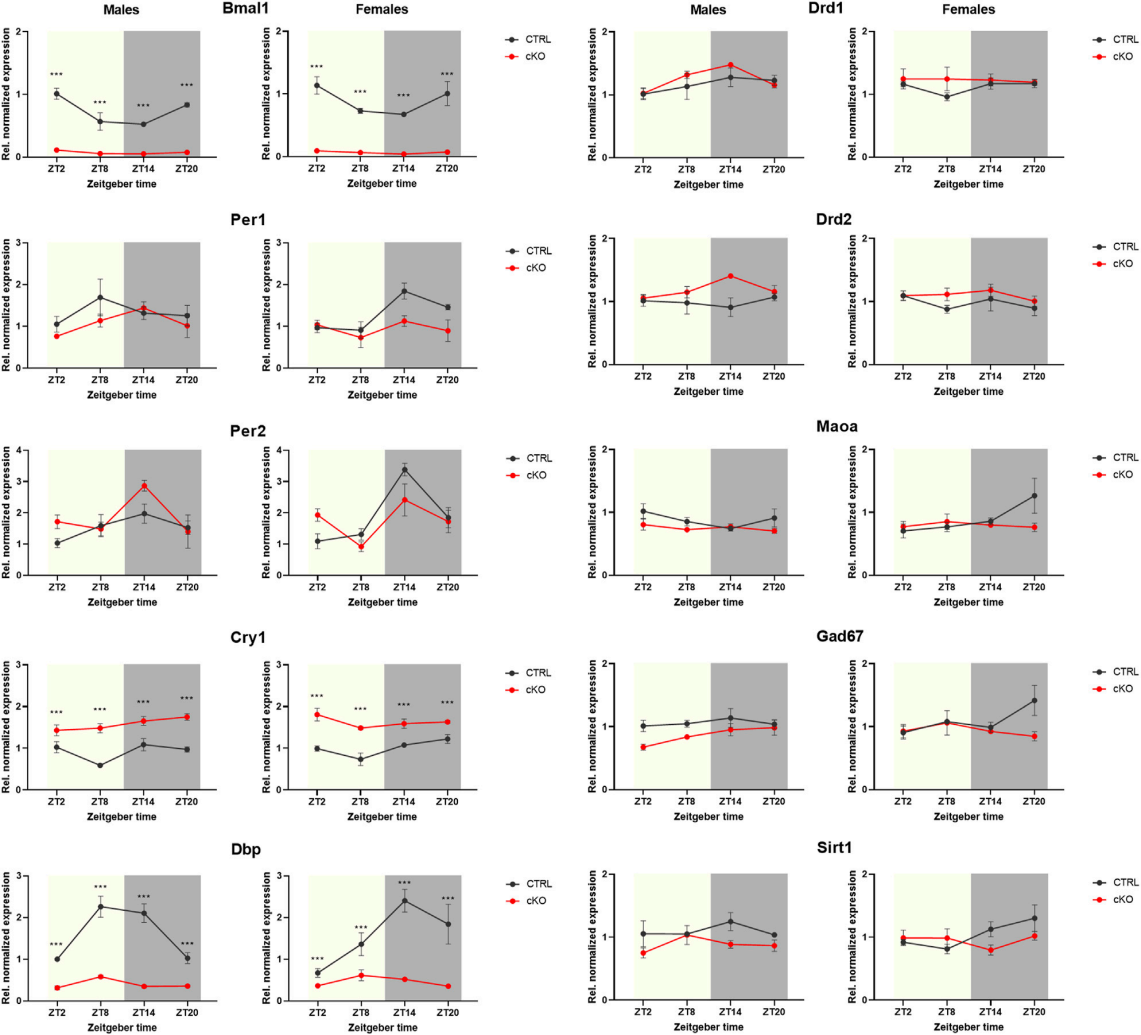
Gene expression analysis of striatal tissue collected from mice with a conditional *Bmal1* knockout and control animals (n = 3–4 per genotype, sex, and time point). While expression of *Bmal1* was abolished, it did only partially affect the expression of E-box-controlled genes. Whereas *Per1* and *Per2* gene expression remained largely unaffected, *Cry1* expression was upregulated. Despite statistical analysis revealing genotype effects of targets of the dopamine and GABAergic signaling pathways in males, differences appeared to be relatively small between genotypes (for details, see result section and Table 4). Results are depicted as mean ± standard error of the mean (S.E.M.).

Visual inspection of expression profiles of clock and clock-controlled genes in male and female *Bmal1* CTRL animals indicates a relatively robust daily oscillation in most of those targets except for *Cry1* (Figure 6 left panel). Statistical analysis partially supported this view. A significant effect of the time of tissue collection on gene expression was found for all clock- and clock-controlled genes (Table 4), however, the zero-amplitude test following cosinor analysis failed to detect significant rhythmicity in most targets. The conditional ablation of *Bmal1* from MSNs, on the other hand, affected the expression of clock- and clock-controlled genes significantly (Table 4). Expression of mRNA at the Exon8 locus of the *Bmal1* gene was almost completely abolished, confirming the conditional knockout of *Bmal1*. CTRL animals, in contrast, display a robust oscillation of *Bmal1* mRNA expression at Exon8. Amongst the other clock genes, no difference between cKO and CTRL male and female individuals was found for *Per2*. Expression peaked at ZT14 in both sexes, and the peak appeared to be more pronounced in cKO males compared to CTRL animals. *Cry1* expression, on the other hand, appeared to be constitutively expressed in mice of both genotypes and sex, but upregulated in cKO individuals. The comparative analysis of *Dbp* expression in cKO and CTRL animals suggests that *Bmal1* is required for proper clock function, demonstrated by blunted *Dbp* expression in cKO mice.

**TABLE 4:** Results of the gene expression analysis.

Target gene	Three-way-ANOVA	Zero-amplitude test		
		Sex	Genotype	F-statistics
Bmal1	genotype: F_1,36_ = 366.6, *p* < 0.0005	M	CTRL	n.s.
			cKO	n.s.
	time point: F_3,36_ = 10.91, *p* < 0.0005			
	time point X genotype: F_3,36_ = 7.158, *p* < 0.005	F	CTRL	n.s.
	gender X genotype: F_3,36_ = 4.244, *p* < 0.05		cKO	n.s.
Per1	genotype: F_1,36_ = 8.422, *p* < 0.05	M	CTRL	n.s.
			cKO	n.s. (*p* = 0.051)
	time point: F_3,36_ = 4.063, *p* < 0.05	F	CTRL	n.s.
			cKO	n.s.
Per2	time point: F_3,36_ = 18.65, *p* < 0.0005	M	CTRL	n.s. (*p* = 0.082)
			cKO	n.s.
		F	CTRL	n.s
			cKO	n.s.
Cry1	genotype: F_1,36_ = 148.5, *p* < 0.0005	M	CTRL	n.s.
	time point: F_3,36_ = 7.081, *p* < 0.005			
			cKO	n.s.
		F	CTRL	n.s.
			cKO	n.s.
Dbp	genotype: F_1,36_ = 162.1, *p* < 0.0005	M	CTRL	n.s. (*p* = 0.078)
	time point: F_3,36_ = 14.81, *p* < 0.0005			
	time point X gender: F_3,36_ = 4.147, *p* < 0.05		cKO	n.s.
	time point X genotype: F_3,36_ = 9.624, *p* < 0.0005	F	CTRL	F_2,1_ = 200.58, *p* = 0.05
	time point X gender X genotype: F_1,36_ = 4.018, *p* < 0.05		cKO	n.s.
Drd1		M	CTRL	n.s.
			cKO	F_2,1_ = 311.92, *p* = 0.04
		F	CTRL	n.s.
			cKO	n.s.
Drd2	genotype: F_1,36_ = 8.375, *p* < 0.05	M	CTRL	n.s.
			cKO	n.s.
		F	CTRL	n.s.
			cKO	n.s.
Maoa	genotype: F_1,36_ = 4.879, *p* < 0.05	M	CTRL	F_2,1_ = 5668.01, *p* = 0.009
			cKO	n.s.
		F	CTRL	n.s.
			cKO	n.s.
Gad67	genotype: F_1,36_ = 10.44, *p* < 0.005	M	CTRL	n.s.
			cKO	n.s.
	time point X gender X genotype: F_1,36_ = 2.993, *p* < 0.005	F	CTRL	n.s.
			cKO	n.s.
Sirt1	genotype: F_1,36_ = 6.173, *p* < 0.05	M	CTRL	n.s.
			cKO	n.s.
		F	CTRL	n.s.
			cKO	n.s.

Genotype effects were revealed by ANOVA when comparing the expression of targets involved in dopaminergic or GABAergic signaling, and cell metabolism, particularly in males (Figure 6 right panel, Table 4). However, expression profiles of those targets varied only marginally between cKO and CTRL animals, based on visual estimation.

## Discussion

The impact of a conditional ablation of the circadian clock genes *Bmal1* or *Per2* from MSNs, the principal neurons of the striatum, on various behaviors was assessed in this study. Results were validated in mice of the *Gpr88* line, to rule out that any behavioral phenotypes observed in *Bmal1* or *Per* cKO animals were the effect of *Gpr88* haploinsufficiency. *Grp88* ^*(cre/+)*^ animals lack one functional copy of the *Gpr88* gene but have intact clock gene expression.

### Anxiety-Like Behavior

Displayed anxiety-related behaviors were not uniform regarding the behavioral tests and varied across clock gene knockouts, but also experimental conditions such as the time of testing and sex. Importantly, the loss of one copy of the *Gpr88* gene did not affect anxiety-like behavior in the EPM, OFT and MBT, even though studies revealed a substantial role of *Gpr88* in the regulation of anxiety-related behaviors (Meirsman et al., 2016a; 2016b). Time of day, however, was a major factor in the regulation of anxiety-like behavior assessed in the EPM and OFT in *Gpr88* ^*(cre/+)*^ and *Gpr88* ^*(+/+)*^ male and female mice, suggesting daily variations in those behaviors. This is in line with the results of a study demonstrating circadian variations of the time spent in the open arm of an elevated plus maze and time spent in the center of an open field (Nakano et al., 2016). Interestingly, animals showed opposed anxiety-like behavior depending on the test, similar to the results in our study. It’s been suggested that anxiety-related behaviors have a multidimensional structure. Therefore, assessment in the EPM or OFT may represent a distinct aspect or trait of anxiety-like behavior depending on the test (van Gaalen and Steckler, 2000; Carola et al., 2002; Nakano et al., 2016; de Brouwer et al., 2019), which may be affected differently by the conditional clock gene knockout. *Bmal1* cKO mice displayed increased open arm time irrespective of the time of day, suggesting that the complete deletion of *Bmal1* from the striatum has an anxiolytic effect. In contrast, *Bmal1* cKO displayed reduced center time particularly at the beginning of the dark period, indicating an anxiogenic effect of the knockout.

In general, relatively little is known about the role of *Bmal1* in the control of anxiety-related behaviors. A conditional knockout in the forebrain did not affect anxiety-like behavior in mice (Snider et al., 2016). Downregulation of the nuclear receptor *Rev-Erb-alpha* (*Rev-Erbɑ*), a repressor of *Bmal1* expression, in the NAc had anxiolytic effects in mice when assessed in the light/dark box (Zhao and Gammie, 2018). However, the study did not provide information on whether *Bmal1* expression was affected by the *Rev-Erb alpha* downregulation, thus the role of *Bmal1* in the observed anxiolytic effect remains speculative. Potential mechanisms of *Bmal1* in the control of anxiety-related behavior are poorly understood but may include E-box regulated expression of other clock or clock-controlled genes. Our study revealed an upregulation of *Cry1* expression in *Bmal1* cKO animals. Interestingly, studies find mixed outcomes of a global *Cry1* knockout on anxiety-like behaviors in mice. When assessed in the EPM and OFT, increased anxiety-like behavior was found (De Bundel et al., 2013), whereas it was reduced in the elevated O-maze test (Schnell et al., 2015). Striatal *Per1* and *Per2* expression, on the other hand, remained relatively unaffected by the conditional *Bmal1* knockout in our study, indicating a mechanism that is independent of the *Per* genes is affecting anxiety-like behavior in *Bmal1* cKO animals. Conversely, other work revealed anxiogenic effects of simultaneous downregulations of *Per1* and *Per2* in the nucleus accumbens in mice assessed in the elevated plus-maze and open field (Spencer et al., 2013). In our study, a conditional *Per2* knockout from striatal MSNs revealed no significant effect on anxiety-like behaviors, although a trend towards decreased anxiety-like behavior in male *Per2* cKO mice was observed. A similar result was found in global *Per2* knockout mice assessed in the open field (Russell et al., 2021), whereas assessment of genome-wide deletions of either *Per1* or *Per2* in the elevated plus-maze revealed mixed outcomes on anxiety-like behavior (Spencer et al., 2013). Thus, further work is needed to fully understand the role of E-box-controlled clock genes such as *Per* and *Cry* genes in the regulation of anxiety-like behavior, with specific emphasis on the specific anxiety traits revealed by the respective behavioral tests. Other factors need to be considered as well, such as the effects of the clock genes outside the clockwork. For instance, downregulation of the *Clock* homolog *Npas2* in the NAc had an anxiolytic effect, presumably through GABAergic neurotransmission (Ozburn et al., 2017).

Assessment of anxiety-like behavior in the MBT revealed no effect of a *Bmal1* or *Per2* knockout. However, it is important to note that the MBT may reflect a better assessment of obsessive-compulsory behavior rather than anxiety (Thomas et al., 2009).

Sex had little influence on anxiety-related behaviors in our study. Despite findings suggesting increased anxiety-related behavior in the open field and elevated plus-maze in C57 female mice (Donner and Lowry, 2013), sex-specific effects were prominent in the OFT in *Per2* cKO only. The estrous stage, however, did not affect anxiety-like behavior in females assessed during the light period. Even though studies revealed mixed outcomes on the effect of daytime on anxiety-like behaviors (Richetto et al., 2018; Meseguer Henarejos et al., 2020; Tsao et al., 2022), our results strongly support the view that time of day is a major factor in the regulation of anxiety-like behavior, and the deletion of clock genes in the striatum appears to attenuate this effect.

### Depressive-Like Behavior

Mood and anxiety disorders are common psychiatric illnesses and epidemiological and clinical data point to high comorbidity between depression and anxiety disorders that may reflect shared pathophysiological mechanisms (Kessler et al., 2008; Lamers et al., 2011), including dysfunctional regulation of circadian clock gene expression (Gouin et al., 2010; Lavebratt et al., 2010; Valenzuela et al., 2016). Studies in rodents confirm this view. Downregulation of *Bmal1* expression in the SCN of mice induced depression- and anxiety-like behavior (Landgraf et al., 2016a). Induction of depressive-like behavior, on the other hand, pointed to alterations of daily *Bmal1* and *Per2* expression profiles in the NAc (Logan et al., 2015; Landgraf et al., 2016b). In our study, the conditional ablation of *Bmal1*, but not *Per2*, from striatal MSNs reduced depressive-like behavior assessed in the tail suspension test. The results indicate that loss-of-function of *Bmal1* in the striatum affects depressive-like behavior differently compared to a dysregulation of its daily expression. The exact mechanism, however, remains elusive. Downregulation of *Bmal1* expression in the NAc reduced the expression of *Cry1* and *Cry2*, but did not affect immobility time in the tail suspension test (Porcu et al., 2020). The same study, however, linked reduced DRD1 activation with upregulated CRY1 expression, presumably through interactions of CRY1 with G-proteins. A pivotal role of CRY1 in the control of dopaminergic neurotransmission in the striatum was suggested by others as well (Schnell et al., 2015). The authors of the study, however, suggest that altered dopamine levels may be causal for observed behavioral phenotypes, conceivably through alteration in the expression of monoamine oxidase A (*Maoa*) by *Per2* (Hampp et al., 2008). The conditional ablation of *Per2* from striatal MSNs, however, did not affect depressive-like behaviour in our study. The results of gene expression analysis in our study furthermore indicated that *Maoa* expression appears to be largely unaffected in *Baml1* cKO mice, thus other factors in the control of depressive-like behaviors must be considered. Daily differences in the expression of the dopamine receptor type 3 (DRD3), which is involved in the regulation of mood and anxiety-like behavior (Moraga-Amaro et al., 2014) were controlled by circadian clock components *retinoic acid receptor-related orphan receptor alpha* (*Rorɑ*) and *Rev-Erbɑ* (Ikeda et al., 2013). Expression of *Rorɑ* and *Rev-Erbɑ* is controlled by *Bmal1* (Preitner et al., 2002), thus future studies should elaborate this link in more detail. One limitation of our study is the assessment of depressive-like behavior at one time point only. Studies indicate that other measures of depressive-like behavior, like anhedonia in the sucrose-preference test, vary over the course of the day (Liu et al., 2018). Our unpublished results in C57BL/6 mice confirm this view. However, a sucrose-preference test conducted in mice of the *Bmal1* and *Per2* lines revealed no difference in sucrose preference between the genotypes (de Zavalia et al., 2021).

### Motor Function

Besides the impact of striatal *Bmal1* and *Per2* deletion on affective behaviors, pronounced effects on motor functioning were found. Despite previous work demonstrates that locomotor activity levels and circadian functioning were not affected by the conditional knockout of *Bmal1* and *Per2* when locomotor activity rhythms were assessed using running wheels (de Zavalia et al., 2021), cKO mice of both strains displayed increased levels of locomotor activity compared to CTRL animals in the open field test. Although studies suggest a role of circadian clock genes in the adaptation to novel environments (Kondratova et al., 2010), novelty-induced hyperactivity in the OFT was likely not caused by a striatal loss-of-function of the respective clock genes in our study. Like *Bmal1* and *Per2* cKO mice, *Gpr88* ^*(cre/+)*^ mice had elevated levels of activity compared to *Gpr88* ^*(+/+)*^ controls. Therefore, hyperlocomotion could be a consequence of a haploinsufficiency of the *Gpr88* gene, which is in line with studies demonstrating locomotor hyperactivity in *Gpr88* knockout animals (Quintana et al., 2012; Meirsman et al., 2016a).

Deficits in motor coordination, on the other hand, were only found in *Bmal1* cKO animals, which show poorer performance in the HBT and FSRR tests. The effects were associated with a loss of *Bmal1* in striatal MSNs. Although it is well known that a knockout of *Gpr88* affects motor function (Quintana et al., 2012; Meirsman et al., 2016a, 2019), no differences were found between *Gpr88* ^*(cre/+)*^ and *Gpr88* ^*(+/+)*^ mice. To our knowledge, this is the first study describing a striatal-specific role of circadian clock genes in the regulation of motor functions. Motor impairment in the rotarod task has been demonstrated in global *Cry1*/*Cry2* knockout mice, and the authors argued that it may be a consequence of elevated anxiety-like behavior observed in those animals (De Bundel et al., 2013). However, this seems unlikely to be the cause of impaired motor coordination in *Bmal1* cKO mice in our study, given the effects on anxiety-like behavior discussed above. Because motor phenotypes resemble symptoms observed in neurodegenerative diseases like Parkinson’s and Huntington’s disease (Carter et al., 1999; Sedelis et al., 2001), we tested whether dopamine signaling was affected by the conditional *Bmal1* knockout. Whereas no difference in the locomotor response following a systemic administration of the dopamine receptor type 2 agonist quinpirole was observed between *Bmal1* cKO and CTRL animals, *Bmal1* cKO mice tended to show a delayed and attenuated response to administration of dopamine receptor type 1 agonist SKF-81297, particularly in females. However, a similar response was observed in male and female *Gpr88* ^*(cre/+)*^ mice compared *Gpr88* ^*(+/+)*^ controls, indicating that a dose-dependent effect of the *Gpr88* gene mitigates the response to DRD1 agonist treatment in the *Gpr88* and *Bmal1* line despite a lack of a significant genotype effect in males and females. Indeed, gene-dosage effects of *Gpr88* on DRD1 and DRD2 function were reported recently (Laboute et al., 2020). The study shows a dose-dependent activation of DRD1 or inhibition of DRD2 receptor activity by *Gpr88,* respectively, mediated through interactions with G-proteins. Therefore, it is conceivable that a lack of one copy of *Gpr88* in *Gpr88* ^*(cre/+)*^ animals contributes to the attenuated response to SKF-81297. Interestingly, the diminished effect of SKF-81297 was more pronounced in *Bmal1* cKO females compared to *Gpr88* ^*(cre/+)*^ females, whereas males of both lines showed a similar response. This suggests that sex-dependent effects of the *Bmal1* knockout add to the observed motor deficits as well. Sex-dependent differences in striatal dopamine receptor expression and regulation of dopaminergic signalling have been described in the literature (Williams et al., 2021; Zachry et al., 2021). Gene expression analysis of targets involved in dopamine signalling pathway, however, revealed no major differences between genotypes and sexes, ruling out the possibility that the *Bmal1* deletion affects expression of targets involved in dopaminergic signalling. However, indirect effects of the *Bmal1* ablation from MSNs could contribute to the attenuated response to SKF-81297. As described above, *Bmal1* cKO mice have increased levels of *Cry1* expression in MSNs, and elevated levels of CRY1 protein may contribute to inhibitory effects on DRD1 functioning (Porcu et al., 2020).

### Striatal Mitochondrial Respiration

It has been suggested that dysfunctions in striatal mitochondrial metabolism may contribute to the development of neurodevelopmental disorders and neurodegenerative disease, which are characterized by motor abnormalities, among other behavioral alterations (Roze et al., 2010; Lu et al., 2018; Wang et al., 2022). Because circadian clock genes, and *Bmal1* in particular, play a pivotal role in the regulation of metabolic processes and have been linked to the development of oxidative stress and neurodegeneration (Musiek et al., 2013; Carter et al., 2021), we compared striatal mitochondrial respiration between *Bmal1* cKO mice and controls. We found no differences between the genotypes in male and female mice, suggesting the behavioral phenotypes in *Bmal1* cKO mice are not linked to mitochondrial respiration, which is in line with findings of other studies (Hamilton et al., 2017). Expression of *Sirt1*, a deacetylase that acts as a metabolic sensor through its NAD^+^ - dependent enzymatic activity, on the other hand, differed between *Bmal1* cKO mice and controls, even though differences were small and likely not of functional significance. *Sirt1* plays a vital role in a variety of metabolic processes such as mitochondrial respiration but also links metabolic homeostasis with circadian clock function (Xu et al., 2018). *Bmal1* cKO and CTRL mice did not display differences in mitochondrial respiration measured at two different time points, suggesting stable metabolic function throughout the day. However, future work should incorporate respirometry measurements during the active phase of the mice to provide a full picture. Interestingly, coupling efficiency was increased in mice of the *Bmal1* line when compared to mice of the *Gpr88* line, presumably through reduced proton leakage in *Bmal1* cKO and CTRL mice, indicating that the deletion of one copy of *Bmal1* in the striatum is sufficient to cause these alterations. Conversely, a study on embryonic stem cells with a *Bmal1* knockout revealed higher respiration accompanied by increased proton leakage and increased mitochondrial ROS production (Ameneiro et al., 2020). The effects, however, may be linked to a specific role of *Bmal1* on cell functions during embryogenesis. Although measurements of ROS levels and oxidative stress responses should be considered in follow up studies, it appears that a deletion of *Bmal1* has only minor effects on mitochondrial respiration in MSN.

### Gene Expression

Mixed outcomes on the effects of gene expression were found in our study. Besides the pronounced effects of the *Bmal1* knockout on *Dbp* expression, other E-box-controlled genes appeared to be less affected, particularly *Per1* and *Per2*. It’s thus tempting to speculate that the expression of those targets is controlled by a mechanism independent of the circadian clock that may contribute to extra-SCN oscillator functioning. Indeed, previous studies found a link between dopamine signaling and clock gene expression (Imbesi et al., 2009; Hood et al., 2010). The strength of this relation, however, needs to be investigated in follow-up studies.

### Limitations

The conditional knockout of clock genes in our study is restricted to MSNs exclusively. Clock gene expression in cells of the striatal network other than MSNs, such as astrocytes, should not be directly affected by the conditional knockout as *Gpr88* is expressed in MSNs only (Massart et al., 2009). A separation between striatal astrocyte and MSN circadian clock function, however, is important as recent work demonstrates that the circadian clock in astrocytes of the NAc contributes significantly to the regulation of behavior, as well as striatal cell metabolism and neurotransmitter signaling (Becker-Krail et al., 2022). This is in line with emerging evidence showing a functional significance of the astrocyte circadian clock on the regulation of daily rhythms (Brancaccio et al., 2019), and the role of striatal astrocytes in governing MSN functions in general (Khakh, 2019). Because anxiety-like behaviors are mildly affected by the conditional *Bmal1* and *Per2* knockout in our study, it is tempting to speculate about a contributing or compensatory role of striatal astrocytes with functional circadian clocks on behavioral control. Likewise, the distinct role of astrocyte clocks on metabolism as well as the impact of astrocyte mitochondrial metabolism on the regulation of metabolic processes in neighboring neurons (Rose et al., 2020; Barca-Mayo and López, 2021) may have affected the results of our measurements on mitochondrial respiration. Although *Bmal1* plays a significant role in cellular metabolism as described above, the effect of a conditional knockout may be compensated by astrocytes with intact clock function and thus explain similar respiration rates in *Bmal1* cKO and CTRL animals observed in our study. Alternatively, the use of whole striatal tissue including clock-intact astrocytes and clock-disrupted MSNs may have contributed to a reduction of recognizable differences between genotypes. The same limitation applies to the measurements of gene expression in our study. Expression profiles of clock genes *Bmal1* and *Per2*, amongst others, in NAc astrocytes displayed profound diurnal rhythms, with identical phases as observed in our study (Becker-Krail et al., 2022). Even though the astrocyte-specific portion on daily gene expression profiles obtained from whole striatal tissue punches of *Bmal1* cKO and CTRL animals in our study cannot be conclusively determined, it is conceivable that it reflected in our gene expression analysis to some extent. Thus, further studies are required to decipher clock-dependent mutual interactions between astrocytes and MSNs in the striatum on behavior, cell metabolism and molecular functioning in more detail.

Overall, the study revealed mild effects of a conditional knockout of *Bmal1* and *Per2* from striatal MSNs on affective behaviors, which was more pronounced in *Bmal1* cKO mice. Deficits in motor coordination, on the other hand, were only found in *Bmal1* cKO animals. Interestingly, anxiety and depression are common comorbidities in neurodegenerative diseases associated with motor dysfunctions (Aarsland et al., 2009). Whether neuropsychiatric and neurodegenerative conditions share a common *Bmal1*-dependent pathophysiological mechanism in MSNs remains elusive, however, the results of our study suggest an interaction of *Bmal1* with the direct dopaminergic signaling pathway as a potential cause. Because of the partially opposing effects of a conditional ablation of *Bmal1* from MSNs on the respective disease states, this would have important implications on the treatment of symptoms associated with those disorders (Eskow Jaunarajs et al., 2011). Future studies are therefore indispensable to elaborate on this link in more detail.

## Data Availability

The raw data supporting the conclusion of this article will be made available by the authors, without undue reservation.
