# Supplementary figures and images for: Characterization of Affective Behaviors and Motor Functions in Mice With a Striatal-Specific Deletion of Bmal1 and Per2
Source: Front Physiol. 2022 Jun 8;13:922080. doi: 10.3389/fphys.2022.922080 (PMC9216244; doi:10.3389/fphys.2022.922080)

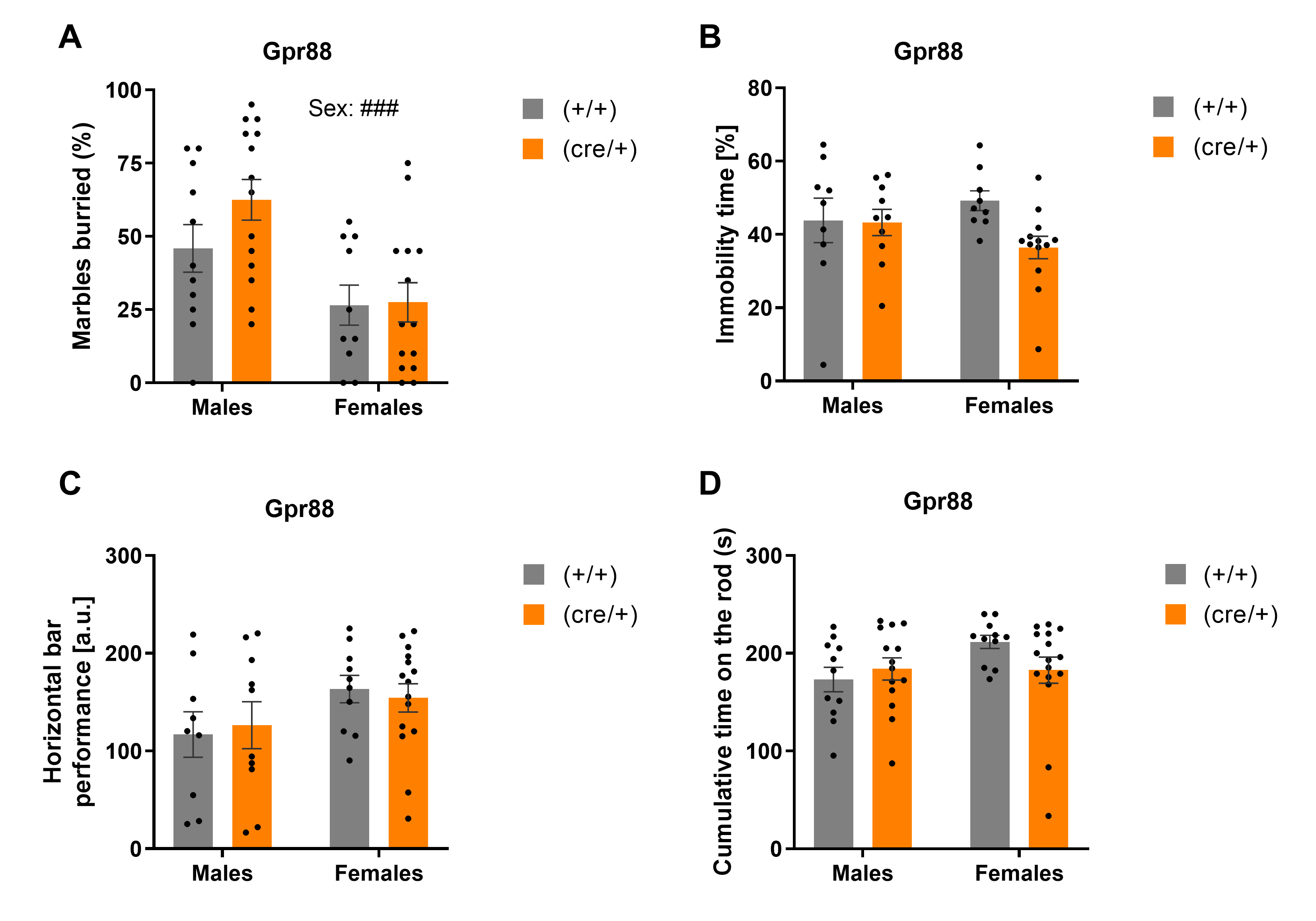

Supplement: Supplementary file 1 [file Image3.TIF]

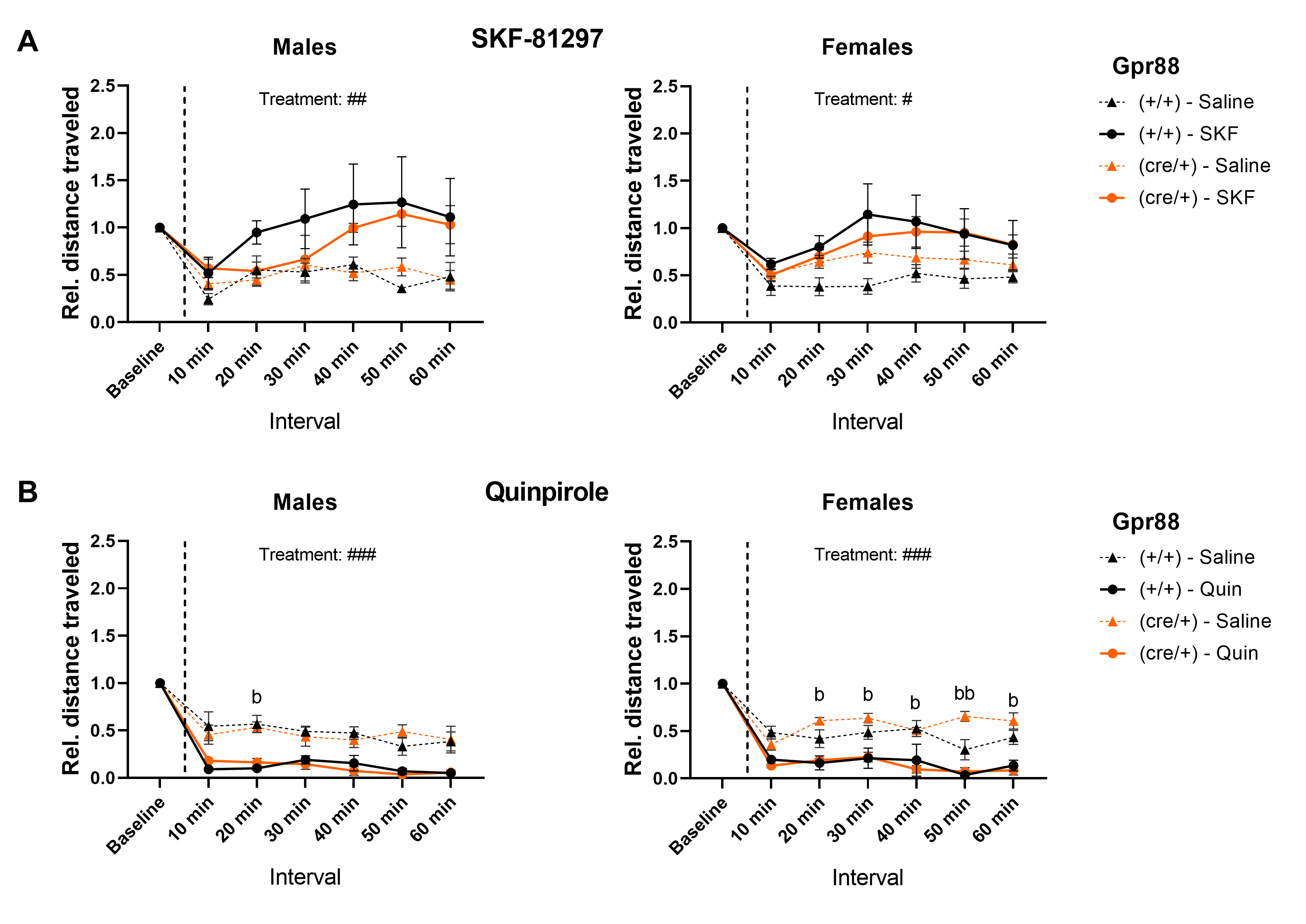

Supplement: Supplementary file 2 [file Image4.TIF]

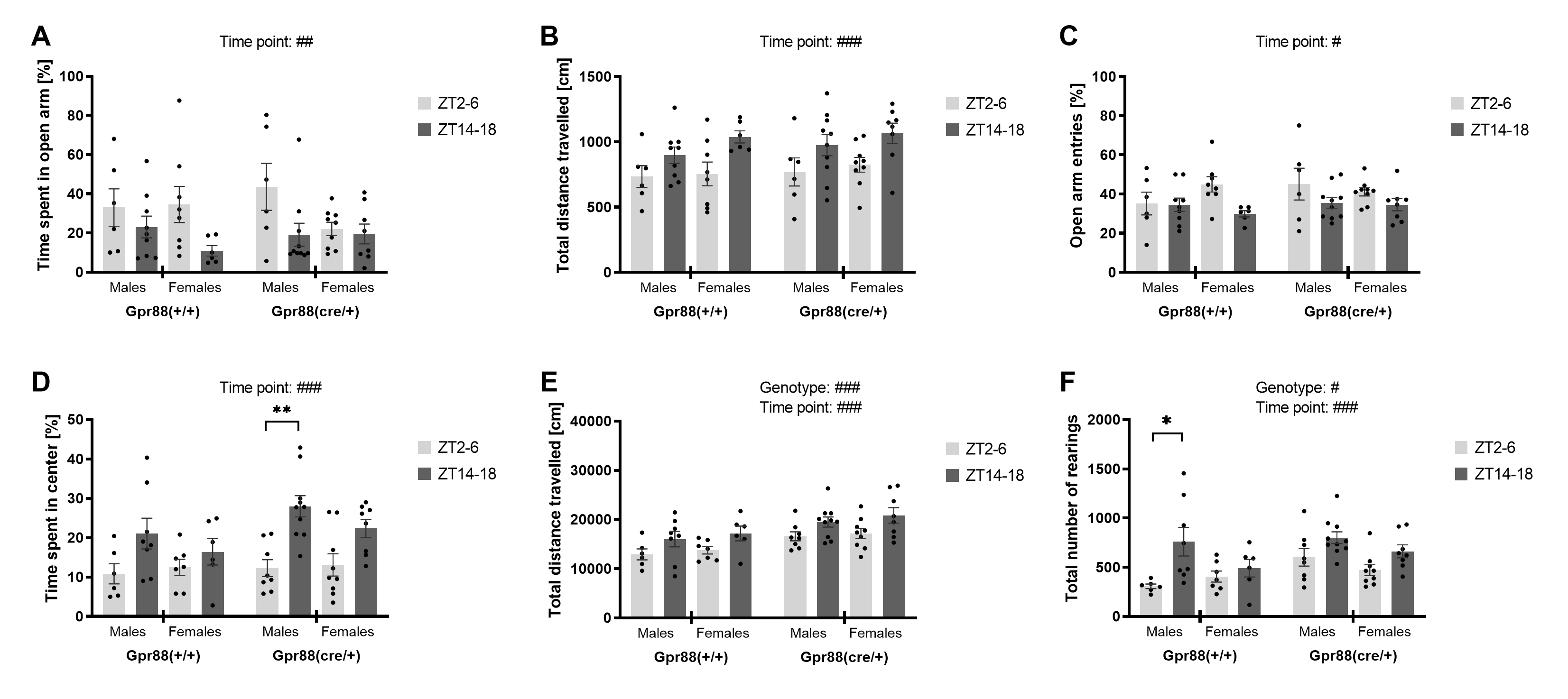

Supplement: Supplementary file 3 [file Image2.TIF]

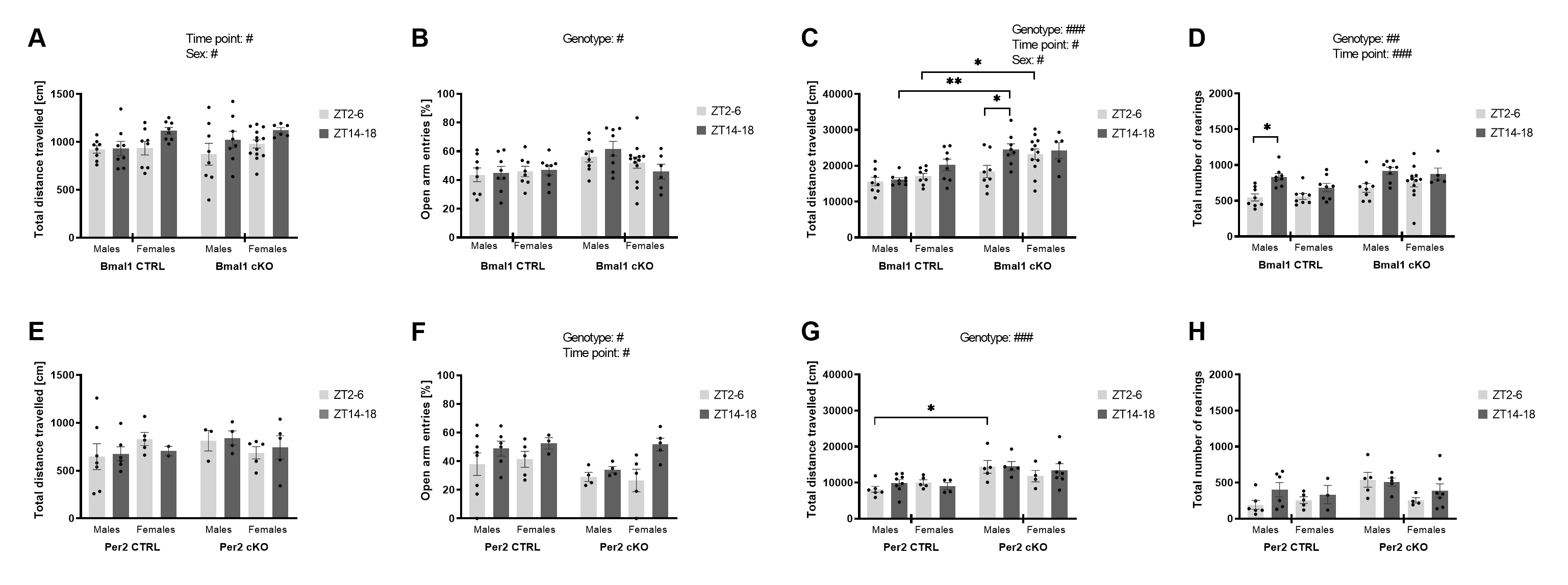

Supplement: Supplementary file 4 [file Image1.TIF]

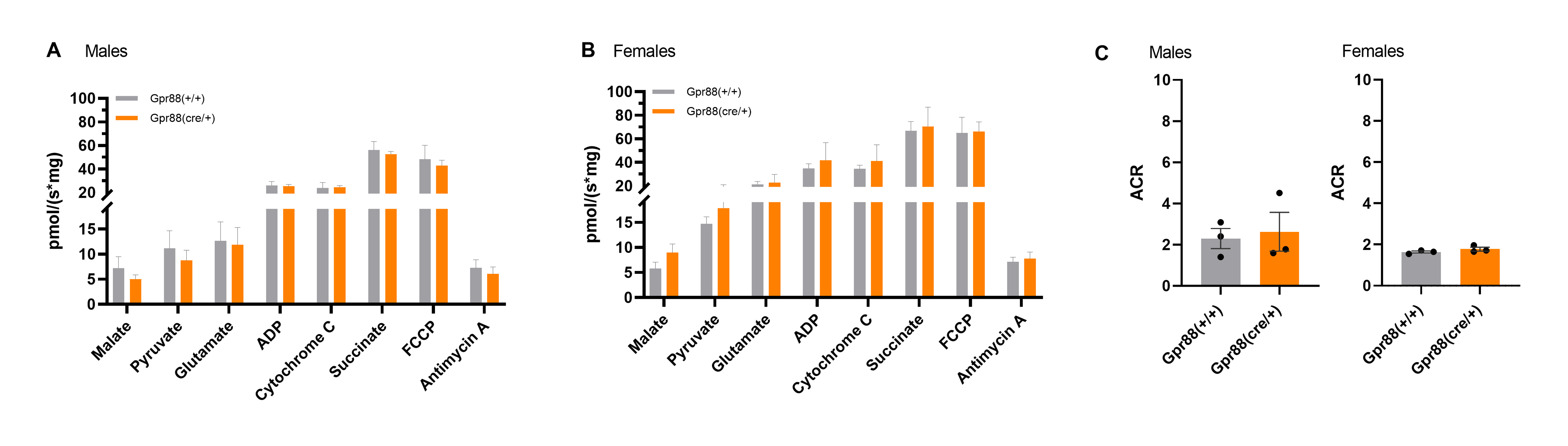

Supplement: Supplementary file 5 [file Image5.TIF]
