# Supplementary material for: Characterization of Affective Behaviors and Motor Functions in Mice With a Striatal-Specific Deletion of Bmal1 and Per2
Source: Front Physiol. 2022 Jun 8;13:922080. doi: 10.3389/fphys.2022.922080 (PMC9216244; doi:10.3389/fphys.2022.922080)
Supplement: Supplementary file 6 [file DataSheet1.DOCX]

Supplementary Figure 1: Activity levels of mice with a striatal-specific clock gene knockout and controls assessed in the elevated plus-maze and open field during the light (ZT2 – 6, light grey bar) and the dark period (ZT 14 – 18, dark grey bar). Whereas total distance travelled in the elevated plus-maze did not differ between genotypes of the *Bmal1* line (n = 6 – 13 per genotype, sex, and time point) (A), the portion of open arm entries was higher in *Bmal1* cKO animals compared to controls (B). Activity levels in the open field were higher in *Bmal1* cKO mice compared to controls. *Bmal1* cKO animals displayed higher horizonal (C) and vertical activity levels (D). Total distance travelled in the elevated plus-maze was not affected by a striatal-specific deletion of *Per2* (n = 3 – 8 per genotype, sex, and time point) (E), whereas the portion of open arm entries lower in *Per2* cKO mice (F). *Per2* cKO animals displayed increased horizontal (G) but not vertical activity levels (H) in the open field. Results are depicted as mean ± standard error of the mean (S.E.M.). # … p ≤ 0.05, ## … p ≤ 0.005, ### … p ≤ 0.0005, three-way ANOVA. * … p ≤ 0.05, ** … p ≤ 0.005, Šídák's multiple comparisons test.

Supplementary Figure 2: Anxiety-like behavior and activity levels in mice of the *Gpr88* line investigated in the elevated plus-maze and open field during the light (ZT2 – 6, light grey bar) and the dark period (ZT 14 – 18, dark grey bar). No genotype-specific differences in anxiety-like behavior and activity levels were observed in mice of the *Gpr88* line (n = 6 – 10 per genotype, sex, and time point) when assessed in the elevated plus maze (A-C). Also, anxiety-like behaviour assessed in the open field was not affected by the loss of one copy of *Gpr88* (D), whereas activity levels were elevated in *Gpr88^(cre/+)^* compared to controls (E,F) indicating that *Gpr88* plays a significant role in the development of a hyperactive phenotype in the open field (n = 6 – 10 per genotype, sex, and time point). Results are depicted as mean ± standard error of the mean (S.E.M.). # … p ≤ 0.05, ## … p ≤ 0.005, ### … p ≤ 0.0005, three-way ANOVA.. * … p ≤ 0.05, ** … p ≤ 0.005, Šídák's multiple comparisons test.

Supplementary Figure 3: Anxiety- and depressive-like behavior and motor functions in mice of the *Gpr88* line (n = 9 – 16 per genotype and sex) were not affected by deletion of one copy of *Gpr88* (A-D). Results are depicted as mean ± standard error of the mean (S.E.M.). ### … p ≤ 0.0005, three-way ANOVA.

Supplementary Figure 4: Locomotor response to DRD1 and DRD2 agonist administration in mice with a deletion of one copy of *Gpr88* and controls. Changes of activity relative to baseline levels are depicted in 10-min intervals. Response of male and female *Gpr88^(cre/+)^* (n = 3 – 8 per sex and treatment) to SKF-81297 treatment was similar compared to controls (n = 4 – 6 per sex and treatment) (A). Activity was inhibited in male and female mice *Gpr88^(cre/+)^* (n = 5 per sex and treatment) and controls (n = 3 – 5 per sex and treatment) following Quinpirole administration compared to saline injections (B), no difference between genotypes were found. Results are depicted as mean ± standard error of the mean (S.E.M.). Only genotype and treatment main effects are displayed in the graph. # … p ≤ 0.05, ## … p ≤ 0.05, ### … p ≤ 0.0005, three-way ANOVA. Letters indicate levels of statistical difference between genotype and treatment post-hoc comparison: b … treatment effect cKO group; one letter … p ≤ 0.05, two letters … p ≤ 0.005, Tukey's multiple comparisons test.

Supplementary Figure 5: Striatal mitochondrial respiration in mice *Gpr88* line. Sequential substrate addition to assess mitochondrial coupled and uncoupled oxygen consumption, as well as leak and membrane integrity, revealed no differences between male and female *Gpr88^(cre/+)^* mice and *Gpr88^(+/+)^* controls (n = 3 per genotype and sex) (A+B). Likewise, the degree of coupling between oxidation and phosphorylation did not differ between genotypes (C). Results are depicted as mean ± standard error of the mean (S.E.M.).
